# Oral Health Knowledge, Attitude, and Behavior of Nursing and Technical Students in Croatia

**DOI:** 10.1055/s-0041-1731852

**Published:** 2021-08-24

**Authors:** Tomislav Cabov, Ksenija Eljuga, Petra Nola Fuchs, Maja Kinkela Devcic, Jelena Prpic, Zoran Kovac, Zrinka Puharic, Irena Glazar, Mirna Zulec

**Affiliations:** 1 Department of Oral Surgery, Faculty of Dental Medicine, University of Rijeka, Rijeka, Croatia; 2 Professional Study Nursing, Bjelovar University of Applied Sciences, Bjelovar, Croatia; 3 Department of Oral Medicine and Periodontology, Faculty of Dental Medicine, University of Rijeka, Rijeka, Croatia; 4 Department of Prosthodontics, Faculty of Dental Medicine, University of Rijeka, Rijeka, Croatia

**Keywords:** attitude, habits, knowledge, oral health

## Abstract

**Objective** The aim of this study was to test knowledge, attitudes, and behavior of the students from the Bjelovar University of Applied Sciences in relation to oral health, and to determine the respectable differences between students of nursing and technical study programs.

**Materials and Methods** Students were randomly selected to represent a convenient sample. A total of 140 students from nursing and technical studies were interviewed by using the World health organization Oral Health Questionnaire, adapted to fit the study purposes.

**Statistical Analysis** Descriptive statistics were used to determine frequency distribution and percentages for all variables. Pearson’s Chi-square test was used to compare the proportions. A *p* -value less than 0.05 was considered to be statistically significant.

**Results** The distribution of participants regarding gender was significantly different between the study programs ( *p* = 0.000). Significant difference was also observed in their perceived socioeconomic status ( *p* = 0.001). A significant difference was found between the study programs regarding the knowledge whether bad teeth could impair general health ( *p* = 0.001), could tooth decay and periodontal disease may be prevented ( *p* = 0.002), as well as the importance of regular dental check-ups for prevention of tooth decay ( *p* = 0.028). There were significant differences regarding dietary habits and alcohol consumption in the past 30 days between the observed study programs ( *p* = 0.000) while no significant differences were found regarding tobacco and tobacco product use ( *p* = 0.170).

**Conclusion** Results obtained and presented in this paper show better knowledge and more favorable habits and behaviors among the students from nursing study program compared with the technical ones. Still all students’ habits should be changed to improve oral health. To change attitudes and influence habits, effective oral health promotion programs are needed; not only in colleges, but also in primary and secondary schools.

## Introduction

World health organization (WHO) describes oral health as a state of complete well-being and functional ability of the teeth and their supporting tissues required for chewing, talking, and esthetics. 1 It has been acknowledged that oral health is of great importance for general health. Numerous investigations and a large body of scientific evidence show the relationship between oral health and some systemic diseases such as cardiovascular disease, diabetes, obesity, arthritis, mild cognitive impairment, and even some malignant diseases. 2 3 Furthermore, oral health is an important indicator of the overall health and well-being, as well as the quality of life. 4 The most common problems seen in the oral cavity are tooth decay (caries), periodontal diseases, and oral carcinoma. One investigation found that oral diseases were present in 3.9 billion people in the world and were led by tooth decay. 5 Periodontal diseases were found in approximately 20% of the world population, while oral carcinoma placed sixth on the list of most common carcinomas in the world. 6 7 Risk factors associated with oral disease development are related to the lifestyle. All of the above mentioned oral conditions may be related to behavior, attitudes, and knowledge on oral health. Food rich in carbohydrates, tobacco, and alcohol consumption; poor oral hygiene and inadequate dental maintenance contribute to the development of oral diseases. 4 6 7 Behavior is defined as a constant and usual mode of carrying out something or behaving and is extremely hard to give up, while the attitude is defined as the way in which a person views and evaluates something or someone. Attitude determines our tendencies of behaving in a certain way. 8 9 Both behavior and attitudes are acquired during one’s life and as such may have either positive or negative effect. However, they may be changed and modified by acquisition of knowledge, in this case on the importance of oral health. Many epidemiological investigations demonstrate a connection between environmental and behavioral factors, which may have detrimental effects on health and lead to development of oral diseases. 10 11 Tooth decay and periodontal diseases are considered as behavioral diseases and may be controlled by altering or modifying certain aspects of lifestyle, in particular adequate maintenance of oral hygiene. Added sugars in food and alcoholic beverages pose the greatest threat related to nutritional habits since they can serve as the energy source for bacteria helping them grow, building plaque deposits, and acids which in turn damage tooth enamel and dentine. 12 Moreover, numerous investigations confirmed that reduced intake of alcoholic beverages and reduction of tobacco consumption may significantly reduce the risk of oral carcinoma development. 13 14 15 Knowledge of the importance of oral health and risk factors for development of oral diseases may have a strong effect on one’s behavior and attitudes, with students being the perfect target group since theirs is the very age when most of the behavior and attitudes are defined. 14 15 16 17 It is feasible that students in healthcare sector will have greater scope of knowledge on health issues, since their very choice of future profession means that they are aware of the importance of not only human health, but also the need to participate actively in providing help to others.

The aim of this study was to test knowledge, attitudes, and behavior of the students from the Bjelovar University of Applied Sciences in relation to oral health, and to determine the respectable differences between students of nursing and technical study programs.

## Materials and Methods

This study was conducted at the Bjelovar University of Applied Sciences, Croatia during the academic year 2018/19. Students were randomly selected from the group of freshmen and sophomores of the nursing and technical programs. A total of 140 students were recruited, 70 from each branch. In both study programs, half of the participants were freshmen and half were sophomores. The mean age of all students was 22.79 ± 6.23 years.

For the purpose of this investigation, the WHO Oral health survey questionnaire was used, with added questions related to the participants’ attitude on oral health. 18 Before filling out the questionnaire, the participants were informed of the aims and purpose of the investigation and signed the informed consent. The consent form and questionnaire were in no way matched. Therefore, the available data could not be correlated to the participants. The participation was entirely voluntary, and regardless of having been selected randomly, the participants were able to drop out of the investigation at any time. This investigation has been approved by the Ethics Committee of the Bjelovar University of Applied Sciences and the Board for biomedical trials of the Faculty of Health Studies in Rijeka, Croatia.

The questionnaire contained questions referring to general information (age, gender, faculty affiliation, study year, and perceived financial status of the family), oral health information (How many natural teeth, crowns, and implants do you have?/In the past 12 months, did your tooth or mouth problems cause any pain or discomfort?/Do you have any removable dentures?/How would you describe the condition of your teeth and gums? Is it “excellent,” “very good,” “good,” “average,” “poor,” or “very poor”?), as well as habits and attitudes on oral health (do you believe bad teeth can impair general health?/How often do you clean your teeth?/Do you use any of the following to clean your teeth/Do you use toothpaste to clean your teeth?/Do you use toothpaste that contains fluoride?/How long has it been since you last saw a dentist?/What was the reason of your last visit to the dentist?/Because of the condition of your teeth or mouth, how often have you experienced any of the following problems in the past 12 months?/How often do you eat or drink any of the following foods, even in small quantities?/How often do you use any of the following types of tobacco/During the past 30 days, on the days you drank alcohol, how many drinks did you usually drink per day?).

## Statistical Analysis

Statistical analysis was performed by using the Statistical Package for Social Science (SPSS). Descriptive statistics were used to determine frequency distribution and percentages for all variables. Pearson’s Chi-square test was used to compare the proportions, and Fisher’s exact test was adopted if necessary. A *p* -value less than 0.05 was considered to be statistically significant.

## Results

A total of 140 students participated in the study, that is, 35 per each study program and study year. Table 1 demonstrates the distribution of participants regarding gender with significant difference between the study programs: 84.4% males in technical programs versus 25.7% in nursing ( *p* = 0.000). Significant difference was observed in their perceived socioeconomic status, where 17.2% of students from technical programs viewed their standing as above average versus only 1.4% of nursing students ( *p* = 0.001).

**Table 1:** Sociodemographic data of the participants

		Technical studies (%)	Nursing program (%)	Total (%)	*p* -Value
Gender	Male	84.4	25.7	53.7
	Female	15.6	74.3	46.3
	Total	100.0	100.0	100.0
Gender	Male	84.4	25.7	53.7
	Female	15.6	74.3	46.3
	Total	100.0	100.0	100.0
Study year	Freshman	54.7	50.0	52.2	0.587
	Sophomore	45.3	50.0	47.8	
	Total	100.0	100.0	100.0	
How would you rate your socioeconomic status?	Below average	4.7	0.0	2.2	0.001
	Average	78.1	98.6	88.8	
	Above average	17.2	1.4	9.0	
	Total	100.0	100.0	100.0	

Regarding the questions on the number of teeth in the mouth, crowns, implants, or dentures, no significant differences were observed between the study groups; the same applies for the questions regarding the condition of teeth and gums.

Oral health attitudes are listed in Table 2 . A significant difference has been found between the study programs regarding the question whether bad teeth could impair general health, where 14.1% of those from technical programs say that they are not sure this is true ( *p* = 0.001). Furthermore, the statistically significant differences were observed regarding the statements: “Tooth decay and periodontal disease may be prevented” ( *p* = 0.002) and “Regular dental check-ups are important for the prevention of tooth decay” ( *p* = 0.028). Students from nursing study program agree significantly more often with these statements compared to the technical students.

**Table 2:** Perceived effect of tooth-related conditions on general health

		Technical studies (%)	Nursing program (%)	Total (%)	*p* -Value **(0.001)**
Decayed teeth cannot affect general health	I agree	82.8	100.0	91.8
	I do not agree	3.1	0.0	1.5
	I am not sure	14.1	0.0	6.7
	Total	100.0	100.0	100.0
Decayed teeth cannot affect general health	I agree	82.8	100.0	91.8
	I do not agree	3.1	0.0	1.5
	I am not sure	14.1	0.0	6.7
	Total	100.0	100.0	100.0
Tooth decay and periodontal diseases are preventable	I agree	81.2	98.6	90.3	0.002
	I do not agree	3.1	1.4	2.2	
	I am not sure	15.6	0.0	7.5	
	Total	100.0	100.0	100.0	
Regular dental check-ups are important for tooth decay prevention	I agree	79.7	94.3	87.3	0.028
	I do not agree	9.4	4.3	6.7	
	I am not sure	10.9	1.4	6.0	
	Total	100.0	100.0	100.0	
I brush teeth regularly to keep them healthy	I agree	87.5	97.1	92.5	0.075
	I do not agree	4.7	0.0	2.2	
	I am not sure	7.8	2.9	5.2	
	Total	100.0	100.0	100.0	
Dental esthetics influences the way others see us	I agree	84.4	92.9	88.8	0.129
	I do not agree	1.6	2.9	2.2	
	I am not sure	14.1	4.3	9.0	
	Total	100.0	100.0	100.0	

The answers to the question (how often do you clean your teeth?) are presented in Table 3 . It is important to note that 46.9% technical students say that they brush twice a day or more often, compared with 78.6% of students from nursing program ( *p* = 0.002). Dental floss is more frequently used by the nursing students ( *p* = 0.021) as it showed at Table 4 .

**Table 3:** Frequency of tooth brushing

		Technical studies (%)	Nursing program (%)	Total (%)	*p* -Value (0.002)
How often do you brush your teeth?	Never	1.6	0.0	0.7	Once a month
	0.0	0.0	0.0	2–3 times a week	6.2
	0.0	3.0	Once a week	0.0	0.0
	0.0	2–6 times a week	7.8	4.3	6.0
	Once a day	37.5	17.1	26.9	Twice a day or more
	46.9	78.6	63.4	Total	100.0
	100.0	100.0			

**Table 4:** Comparison between the means for oral health maintenance

Do you use any of the following to clean your teeth?		Technical studies (%)	Nursing program (%)	Total (%)	*p* -Value
Toothbrush	Yes	100.0	100.0	100.0	
	No	0.0	0.0	0.0	
	Total	100.0	100.0	100.0	
Wooden toothpicks	Yes	37.5	27.1	32.1	0.200
	No	62.5	72.9	67.9	
	Total	100.0	100.0	100.0	
Plastic toothpicks	Yes	9.4	8.6	9.0	0.871
	No	90.6	91.4	91.0	
	Total	100.0	100.0	100.0	
Dental floss	Yes	34.4	54.3	44.8	0.021
	No	65.6	45.7	55.2	
	Total	100.0	100.0	100.0	
Chewstick/miswak	Yes	3.1	1.4	2.2	0.507
	No	96.9	98.6	97.8	
	Total	100.0	100.0	100.0	
Other	Yes	10.9	8.6	9.7	0.644
	No	89.1	91.4	90.3	
	Total	100.0	100.0	100.0	

As for the dietary habits, there are significant differences regarding consumption of fruit ( *p* = 0.003), sweet foods ( *p* = 0.007), carbohydrate-rich foods ( *p* = 0.005), and sugar chewing gums ( *p* = 0.010).

Finally, there were no significant differences regarding tobacco and tobacco product use ( *p* = 0.170), whereas alcohol consumption in the past 30 days ( Table 5 ) showed significant differences between the observed study programs ( *p* = 0.000), with 12.5% of the technical students stating that they consume less than one drink per day, versus 38.6% of the students from nursing program.

**Table 5:** Alcohol consumption in the past 30 days

During the past 30 days, on the days you drank alcohol, how many drinks did you usually drink per day?	**Technical** studies **(%)**	Nursing program **(%)**	Total **(%)**	*p* -Value
Less than one	12.5	38.6	26.1	0.000
1 drink	10.9	12.9	11.9	
2 drinks	10.9	11.4	11.2	
3 drinks	10.9	10.0	10.4	
4 drinks	3.1	10.0	6.7	
5 or more drinks	45.3	17.1	30.6	
I have not been drinking any alcohol for the past 30 days	6.2	0.0	3.0	
Total	100.0	100.0	100.0	

## Discussion

The scope of numerous investigations performed around the globe among people belonging to different age groups has been to gain insight into the knowledge, behavior, and attitudes related to oral health. During the past decades, a significant decrease in prevalence of oral diseases has been observed, especially in developed countries, which has been linked to the awareness of the importance of oral health, implementation of healthy lifestyle, and effective application of prevention programs. 19 Young people, including students, are an important segment of the society, since their behavior may play a crucial role in the positive change of behavior reflected in the entire community. 20 21 To increase the effectiveness of the education and prevention programs, one should gain some insight into the knowledge, behavior, and attitudes of this target group. This investigation provided the information on general data, oral health data, attitudes, and behavior regarding oral health in the group of nursing and technical students.

In this study population, there was a statistically significant difference regarding the participants’ gender ( *p* = 0.000). The questionnaire was distributed to 84.4% of males attending the technical programs versus 25.7% in nursing program. Male gender is predominant at the technical programs of the Bjelovar University of Applied Science, whereas the opposite goes for nursing. This result may be explained by the fact that technical study programs are more frequently attended by men, while females are predominant students at biomedical faculties including nursing programs. This tendency has also been reported by Buccheri 22 who confirmed that females are more interested in medical study programs than males who prefer technical knowledge. The interest in programs such as computing and electrotechnics is rising due to great technological advances in those fields and possibility of application of the knowledge in almost every line of work. Furthermore, upon completion, students usually face no problems in finding the job while being offered above the average pay levels. It may therefore be expected that more women will apply to those study programs in the future, and not only in Croatia.

Regarding the question on the students socioeconomic status, significant differences were observed between the study programs. Of those interviewed, 17.2% of the technical students reported their status as above the average compared just 1.4% of nursing students. These results are not in line with the available investigations demonstrating that technical students are usually more financially vulnerable compared with their biomedical counterparts. Al-Hussaini 12 analyzed sociodemographic data on medical, pharmacology, and dental students, and 51.5% of them stated their families had incomes above the average, while 34.9% stated their family income was average.

Table 2 showing the students’ attitudes regarding the effect dental health on overall health also demonstrates significant differences between study groups. All nursing students were affirmative (100%) versus 82.8% of technical students. For comparison, Gopikrishn 23 showed that 73% of the engineering students and 74.8% of their nursing counterparts consider dental health as an important factor in general health. As for the question whether tooth decay and periodontal diseases are preventable, 98% of nursing students said “yes” versus 81.2% of technical students. Furthermore, 94.3% of students attending nursing programs think that regular dental check-ups can prevent tooth decay, while 79.7% of the students from technical programs gave the same answer. Similar data were obtained by Jaramillo 24 who showed that the nursing students had superior knowledge on oral health compared to the students from technical faculties. Nursing students are the future promoters of the importance of health maintenance and oral health knowledge is implemented in their study programs. Finally, the available results demonstrate that nursing students have more positive attitudes towards the importance of oral health compared to the technical ones.

Table 3 presents data related to oral habits. Based on the acquired knowledge, one develops habits performed daily usually without thinking. The question (“how often do you brush your teeth?”) showed significant differences between the observed study programs, 46.9% of students from technical programs stated that they brushed twice a day or more, compared to 78% of those from nursing program who provided the same answer. There are 37.5% of technical students who brush only once a day versus 17.1% of the nursing ones, this difference is significant. Kaira et al 25 tested the knowledge, attitudes, and behavior among the nursing students at the Medical School in India, and they published the results of 111 participants, 48.7% of students brush their teeth twice a day and 28.9% of students brush once a day. Results of the investigation presented hereby demonstrate more nursing students brush twice a day compared to the nursing students in India. In another investigation from Lithuania by Pacauskien, 26 92% students from biomedical colleges brush their teeth twice a day, whereas 73.3% of technical students reported the same habit. If we should compare these results to those hereby presented, students from Croatia demonstrated poorer oral hygiene maintenance habits compared to those from Lithuania.

Dental floss is a valuable addition to oral hygiene maintenance, and it is used by 34.4% of the students from technical study programs compared with 54.3% of the students from nursing program. This difference is statistically significant. For comparison, in Lithuania 44.6% of the biomedical and 17.3% of the technical final year students use dental floss. 26 We may conclude that students from the Bjelovar University of Applied Science used dental floss more often.

The question (when did you last visit your dentist?) shows a marking difference between the observed study groups: 32.8% of the students from technical study programs state less than 6 months compared to 50% of the students from nursing students. Similarly, very interesting results were obtained by Gopikrishna, 23 showing that dental check-ups fail to follow the trends. For example, 28.05% never visit their dentists, while 38.7% visit the dentist only when they feel the pain. Distribution among the students show that 39.1% technical students and 34.3% nursing students visited their dentists only when in pain. As reported in the investigation by Kaira, 25 19.8% of the nursing students visit their dentists twice a year. It has been widely accepted that regular dental check-ups are necessary every 6 months, therefore this reported behavior obviously fails to follow these recommendations.

Considering the observed study programs, there are significant differences regarding dietary habits. The data show that—surprisingly—nursing students have poorer dietary habits. Beside playing an important role in maintaining the general health, healthy and balanced diet may have an important effect on one's intellectual achievements, emphasizing the importance of the right choice of nutrients in everyday life. 27 It also important that all future health professionals have a healthy diet and promote healthy lifestyles. Smoking poses a risk factor for development of numerous oral diseases. According to the available data, the percentage of students who smoke varies significantly from 2.4 to 37%. 28 Pacauskiene et al 26 found that 26.7% of technical students smoke, compared with 9% of biomedical sophomores and 12% of final year students in biomedical programs. Our results showed no differences between students. Another problem among students is alcohol consumption. Changes in life circumstances, lack of parental surveillance, and increased stress lead to an increased intake of alcoholic beverages, leading to many undesired consequences. This investigation found significant differences regarding the observed study groups. Regarding the number of drinks per day, 12.5% of technical students say less than one per day versus 38.6% of nursing ones. This may at least partially be explained by the fact that nursing study programs are more frequently attended by female students. 29

## Conclusion

Results obtained and presented in this paper show better knowledge and more favorable habits and behaviors among the students from nursing study program compared with the technical ones. One of the possible reasons in the fact that nursing study program offer more educational subjects focusing on health and disease prevention, including oral health. Students are therefore educated on the right approach to health and disease, and analyze public health issues.

Oral health is an integral part of the overall health maintenance. Tooth decay is one of the most common oral problems and is considered as a huge public health problem in the whole world. Taking into account a high occurrence of tooth decay in the population, a more intensive general education should be deemed necessary. Students are particularly vulnerable groups, and they should be further educated through workshops. Furthermore, public actions should be taken more frequently, being provided with a greater media access, particularly for the oral health experts. Mandatory routine check-ups must be introduced for the most vulnerable groups, and dental examination should become an integral part of the standard medical examination.

## References

[1] Petersen P E The World Oral Health Report 2003: continuous improvement of oral health in the 21st century–the approach of the WHO Global Oral Health Programme Community Dent Oral Epidemiol 2003 31(1, Suppl 1)3 23 15015736 10.1046/j..2003.com122.x

[2] Sabbah W Folayan M O El Tantawi M The link between oral and general health Int J Dent 2019 7.862923E6 10.1155/2019/7862923 PMC6560319 31275387

[3] Saadeh R Bober-Moken I Challa S Relationship between general health behaviors and oral health behaviors in 2015-2016 NHANES adult population Eur J Dent 2019 13 03 405 412 31618787 10.1055/s-0039-1698364 PMC6890499

[4] Zheng S Zhao L Ju N Hua T Zhang S Liao S Relationship between oral health-related knowledge, attitudes, practice, self-rated oral health and oral health-related quality of life among Chinese college students: a structural equation modeling approach BMC Oral Health 2021 21 01 99 33676475 10.1186/s12903-021-01419-0 PMC7936478

[5] Richards D Oral diseases affect some 3.9 billion people Evid Based Dent 2013 14 02 35 23792391 10.1038/sj.ebd.6400925

[6] AlJehani Y A Risk factors of periodontal disease: review of the literature Int J Dent 2014 2014 182513 24963294 10.1155/2014/182513 PMC4055151

[7] Kumar M Nanavati R Modi T G Dobariya C Oral cancer: etiology and risk factors: a review J Cancer Res Ther 2016 12 02 458 463 27461593 10.4103/0973-1482.186696

[8] Shah A F Batra M Sudeep C B Gupta M Kumar R Oral habits and their implications Ann Med 2014 1 04 179 186

[9] Al-Wesabi A A Abdelgawad F Sasahara H El Motayam K Oral health knowledge, attitude and behaviour of dental students in a private university BDJ Open 2019 5 16 31666985 10.1038/s41405-019-0024-x PMC6813301

[10] Petersen P E Sociobehavioural risk factors in dental caries - international perspectives Community Dent Oral Epidemiol 2005 33 04 274 279 16008634 10.1111/j.1600-0528.2005.00235.x

[11] Petersen P EInequalities in oral health – the social context for oral health. In: Harris R, Pine C, eds. Community Oral Health. London: Quintessence2005

[12] Al-Hussaini R Al-Kandari M Hamadi T Al-Mutawa A Honkala S Memon A Dental health knowledge, attitudes and behaviour among students at the Kuwait University Health Sciences Centre Med Princ Pract 2003 12 04 260 265 12966201 10.1159/000072295

[13] Goldstein B Y Chang S C Hashibe M La Vecchia C, Zhang ZF. Alcohol consumption and cancers of the oral cavity and pharynx from 1988 to 2009: an update Eur J Cancer Prev 2010 19 06 431 465 20679896 10.1097/CEJ.0b013e32833d936d PMC2954597

[14] Gray J L Maghlouth A A Hussain H A Sheef M A Impact of oral and oropharyngeal cancer diagnosis on smoking cessation patients and cohabiting smokers Tob Induc Dis 2019 17 75 31768167 10.18332/tid/109413 PMC6843181

[15] Ashraf N azir M Almas K Awareness about the effects of tobacco consumption on oral health and the possibility of smoking behavior among male Saudi schoolchildren Eur J Dent 2017 11 01 29 35 28435362 10.4103/ejd.ejd_300_16 PMC5379831

[16] Blaggana A Grover V Oral health knowledge, attitudes and practice behaviour among secondary school children in Chandigarh J Clin Diagn Res 2016 10 10 ZC01 ZC06 10.7860/JCDR/2016/23640.8633 PMC5121785 27891447

[17] Tanny L Komabayashi T Long D L Yahata Y Moffat S M Tãne H The effect of education on oral health students’ attitudes in Australia and New Zealand Eur J Dent 2016 10 04 491 495 28042264 10.4103/1305-7456.195178 PMC5166305

[18] World Health Organization Oral health surveys. Basic Methods2013

[19] Zhu L Petersen P E Wang H Y Bian J Y Zhang B X Oral health knowledge, attitudes and behaviour of adults in China Int Dent J 2005 55 04 231 241 16167612 10.1111/j.1875-595x.2005.tb00321.x

[20] Bandyopadhyay A Bhuyan L Panda A Dash K C Raghuvanshi M Behura S S Assessment of oral hygiene knowledge, practices, and concepts of tobacco usage among engineering students in Bhubaneswar, Odisha, India J Contemp Dent Pract 2017 18 06 423 428 28621268 10.5005/jp-journals-10024-2059

[21] Birant S Koruyucu M Ozcan H Investigating the level of knowledge of the community about oral and dental health Eur J Dent 2021 15 01 145 151 32932530 10.1055/s-0040-1716583 PMC7902119

[22] Buccheri G Abt Gürber N Brühwiler C The impact of gender on interest in science topics and the choice of scientific and technical vocations Int J Sci Educ 2011 33 01 159 178

[23] Gopikrishna V Bhaskar N N Kulkarni S B Knowledge, attitude, and practices of oral hygiene among college students in Bengaluru city J Indian Assoc Publ Health Dent 2016 14 01 75 79

[24] Jaramillo J A Jaramillo F Kador I A comparative study of oral health attitudes and behavior using the Hiroshima University-Dental Behavioral Inventory (HU-DBI) between dental and civil engineering students in Colombia J Oral Sci 2013 55 01 23 28 23485597 10.2334/josnusd.55.23 PMC4090926

[25] Kaira L S Srivastava V Giri P Chopra D Oral health-related knowledge, attitude and practice among nursing students of Rohilkhand Medical college and hospital: a questionnaire study J Orofac Res 2012 2 01 20 23

[26] Pacauskiene I M Smailiene D Siudikienė J Savanevskyte J Nedzelskiene I Self-reported oral health behavior and attitudes of dental and technology students in Lithuania Stomatologija 2014 16 02 65 71 25209229

[27] Reuter P R Forster B L Brister S R The influence of eating habits on the academic performance of university students J Am Coll Health 2020 6 1 7 10.1080/07448481.2020.1715986 32027236

[28] Shah A H ElHaddad S A Oral hygiene behavior, smoking, and perceived oral health problems among university students J Int Soc Prev Community Dent 2015 5 04 327 333 26312233 10.4103/2231-0762.161765 PMC4547449

[29] Davoren M P Cronin M Perry I J O’Connor K Alcohol consumption among university students: a typology of consumption to aid the tailoring of effective public health policy BMJ Open 2016 6 11 e011815 10.1136/bmjopen-2016-011815 PMC5128944 27852707

